# Diabetes‐Specific Serum Calcium Thresholds for Mortality Risk: A NHANES Nutritional Epidemiology Study

**DOI:** 10.1002/fsn3.71034

**Published:** 2025-09-23

**Authors:** Ling Li, Shuangyu Yang, Xiao Ran, Sudong Liu

**Affiliations:** ^1^ Institute of Basic Medical Sciences Meizhou People's Hospital (Huangtang Hospital) Meizhou P. R. China; ^2^ Department of Medical Research Center, Sun Yat‐sen Memorial Hospital Sun Yat‐sen University Guangzhou China; ^3^ Department of Nephrology Meizhou People's Hospital (Huangtang Hospital) Meizhou P. R. China; ^4^ Guangdong Engineering Technology Research Center of Molecular Diagnostics for Cardiovascular Diseases Meizhou P. R. China

**Keywords:** mortality risk, NHANES, precision nutrition, serum calcium, type 2 diabetes mellitus

## Abstract

This retrospective cohort study aimed to characterize diabetes‐stratified associations between serum calcium levels and all‐cause, cardiovascular (CVD), and cancer mortality, addressing a critical gap in nutritional epidemiology. Leveraging the National Health and Nutrition Examination Survey (NHANES) database, we conducted Cox proportional hazards regression with restricted cubic splines to model non‐linear calcium–mortality relationships. Mediation analysis was conducted in diabetes. The T2DM group presented a J‐shaped relationship, with relatively stable and low mortality risk at lower calcium concentrations, followed by a significant increase at higher concentrations. In contrast, the group without diabetes exhibited a U‐shaped relationship, indicating that both low and high serum calcium levels are associated with increased mortality risk, with an optimal range in the middle where the risk is minimized. Mediation analysis indicated that, in individuals with T2DM, the relationship between serum calcium levels and mortality was partially mediated by markers of renal function and total cholesterol. Our findings support personalized calcium monitoring as a cost‐effective strategy in diabetic care. Serum calcium thresholds for mortality risk differ fundamentally by diabetes status, underscoring the need for precision nutrition strategies. Integration of calcium monitoring into routine diabetic care—particularly targeting high‐risk subgroups—may mitigate mortality.

## Graphical abstract

This study reveals that diabetes status fundamentally alters the relationship between serum calcium and mortality risk. A J‐shaped risk curve was identified in individuals with type 2 diabetes, contrasting with a U‐shaped curve in the non‐diabetic population, underscoring the need for personalized calcium monitoring in clinical practice.

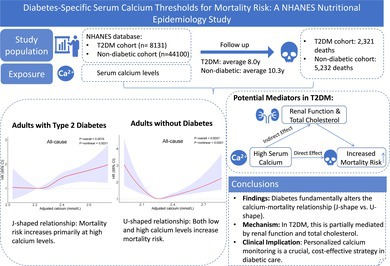

## 1 Introduction

Calcium plays a vital role in various physiological processes (Singh et al. 2018), and its serum concentration is tightly regulated. Abnormal serum calcium levels have been linked to various health conditions such as cardiovascular diseases (CVDs) (Larsson et al. 2017; Qiu et al. 2024; Yang, Miao, et al. 2023), cancers (Wulaningsih et al. 2013; Wulaningsih et al. 2016; Zheng et al. 2023), and metabolic disorders (Becerra‐Tomás et al. 2014).

Type 2 diabetes mellitus (T2DM) represents a significant metabolic disorder that has become a critical global health concern. The global prevalence of diabetes among individuals aged 20–79 years was estimated to be 10.5% (approximately 536.6 million) in 2021, with projections indicating an increase to 12.2% (approximately 783.2 million) by 2045 (Sun et al. 2022). In addition to glycemic control, various metabolic abnormalities in individuals with T2DM contribute to its complications, including cardiovascular disease (Wilcox et al. 2020) and cancer (Giovannucci et al. 2010), which are the leading causes of mortality in these individuals.

Recently, attention has shifted towards elucidating the role of serum calcium levels in the progression of T2DM and its complications. Calcium homeostasis, modulated by dietary sources (e.g., dairy, fortified foods) and endogenous regulatory pathways, critically influences metabolic health. Within pancreatic islets, β‐cell calcium oscillations govern glucose‐stimulated insulin secretion (Roy et al. 2025), while dysregulated Ca^2+^ handling impairs insulin exocytosis and promotes T2DM pathogenesis (Rutter et al. 2024). According to some researchers, serum Ca^2+^ dyshomeostasis is associated with the development of insulin resistance and impaired glucose tolerance (Zhu et al. 2019). Several observational studies have reported a positive association between high serum calcium levels and increased risk of T2DM (Zhai et al. 2023).

Beyond pancreatic dysfunction, aberrant calcium signaling induces vascular damage in T2DM and metabolic syndrome via pro‐calcific pathways and endothelial impairment (Romero‐García et al. 2025; Rubin et al. 2007). A study revealed that serum calcium levels were positively associated with carotid plaque thickness, which is a powerful predictor of CVD events (Rubin et al. 2007). A non‐linear association has been identified between serum calcium levels and CVD mortality, particularly among individuals with chronic kidney disease (CKD) (Yang, Kweon, et al. 2023). Crucially, calcium supplementation demonstrates diabetes‐specific risk differentials, significantly increasing CVD events and mortality in diabetic populations but not in non‐diabetic individuals (Qiu et al. 2024). This evidence collectively establishes serum calcium as a modifiable nutritional determinant for precision nutrition strategies targeting metabolic mortality.

Previous research has revealed significant differences in all‐cause mortality and cause‐specific mortality rates between individuals with diabetes and those without diabetes, along with their trends over time (Gregg et al. 2018). However, the relationship between the serum calcium concentration and mortality in different populations remains unclear. The primary objective of this study was to assess the dose–response relationship between the serum calcium concentration and all‐cause, CVD, and cancer mortality in populations with T2DM and those without diabetes, with the aim of developing the best management strategies for controlling calcium levels to minimize mortality risk in different groups. We further hypothesized that renal and lipid metabolic pathways mediate calcium‐mortality relationships in diabetes—a mechanistic insight with direct implications for nutritional interventions targeting these modifiable factors.

## 2 Methods

### 2.1 Study Subject

The National Health and Nutrition Examination Survey (NHANES) employs a sophisticated multistage probability sampling methodology to derive representative samples from the non‐institutionalized civilian population of the United States. This survey includes health and nutrition interviews, physical examinations, and laboratory tests (Chen et al. 2020; Curtin et al. 2013; Johnson et al. 2014; Johnson et al. 2013). The NHANES has received approval from the Research Ethics Review Board of the National Center for Health Statistics (NCHS). For our analysis, we used data from the continuous NHANES cycles spanning the years 1999–2018, which included 101,316 participants. We excluded 42,112 participants under the age of 18, resulting in a total of 59,204 adult participants. Participants whose serum calcium or serum albumin measurements were missing were also excluded. Additionally, those without follow‐up data were removed from the analysis. Diabetes was defined according to the criteria from a previous study (Gregg et al. 2018). Participants were considered to have diabetes if they met any one of the following conditions:

- Self‐reported diabetes diagnosis by a healthcare professional.
- Use of anti‐diabetic medications or insulin.
- Glycated hemoglobin A1c (HbA1c) level ≥ 6.5% (48 mmol/mol).
- Fasting plasma glucose level ≥ 7.0 mmol/L.
- Two‐hour plasma glucose level ≥ 11.1 mmol/L after an oral glucose tolerance test.

Participants who did not meet any of these criteria were considered participants without diabetes. Individuals potentially having type 1 diabetes (*n* = 274), identified by insulin use and diabetes onset before age 30 (confirmed to be accurate in 97% of cases), were excluded (Li et al. 2022).

**FIGURE 1:**
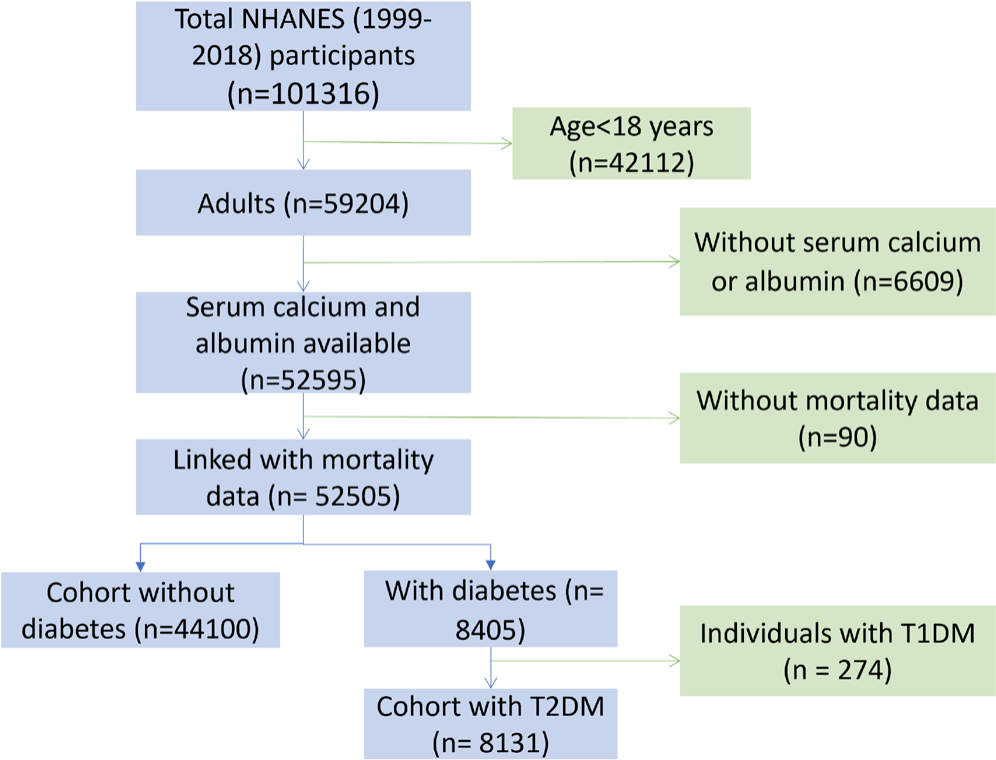
Participant selection flowchart for the analysis of the association between serum calcium levels and mortality risk in cohorts with T2DM or without diabetes. NHANES, National Health and Nutrition Examination Survey; T1DM, type 1 diabetes; T2DM, type 2 diabetes.

Ultimately, the analysis included 8131 participants with T2DM and 44,100 participants without diabetes with available mortality follow‐up data and measured serum calcium concentrations (Figure 1).

### 2.2 Evaluation of Albumin‐Adjusted Serum Calcium

The serum calcium concentrations used in this study were all albumin‐adjusted serum calcium concentrations. Serum total calcium and albumin levels were measured in blood samples collected at mobile examination centers (MECs) via a Beckman Synchron LX20 analyzer. The albumin‐adjusted serum calcium concentration was determined via the following formula (Nigwekar et al. 2013):

$$\begin{array}{c} \text{Serum calcium}\;\left(\text{mmol}/L\right)=\text{total serum calcium}\;\left(\text{mmol}/L\right)\\ \;+0.02^{*}\;\left(40\;g/L-\text{serum albumin}\;\left[g/L\right]\right) \end{array}$$

To ensure an even distribution across the data set, participants in each cohort were stratified into four distinct groups on the basis of the quartiles of their albumin‐adjusted serum calcium levels (Yang, Miao, et al. 2023): Cohort with T2DM: Q1 (1.560–2.270), Q2 (2.270–2.325), Q3 (2.325–2.385), Q4 (2.385–3.225); Cohort without diabetes: Q1 (1.590–2.245), Q2 (2.245–2.300), Q3 (2.300–2.355), Q4 (2.355–3.660).

### 2.3 Assessment of Covariates

Information on demographic and health‐related factors was self‐reported by the participants. These factors include age, sex, race, body mass index (BMI), educational attainment, household income, smoking status, alcohol consumption, medication use, duration of diabetes, and CVD status. BMI was determined by dividing weight in kilograms by the square of height in meters. Racial categories included non‐Hispanic White, non‐Hispanic Black, Mexican American, other Hispanic, and other/multiracial. Educational attainment was classified into four levels: < 9th grade, 9–11th grade, high school, and college or above. Household income was measured as the poverty‐income ratio. Smoking status was categorized into three groups: never smoker, former smoker, and current smoker. Drinking status was categorized into four distinct groups: 0, 1–5, 5–10, and 10+ drinks per month. Participants were considered to have CVD if they had been diagnosed by a healthcare professional. Hypertension was defined as a self‐reported history of hypertension, an average measured blood pressure of ≥ 140/90 mmHg, or the use of anti‐hypertensive medication (Li et al. 2022). Clinical indicators such as fasting glucose, insulin, HbA1c, total cholesterol (TC), low‐density lipoprotein cholesterol (LDL‐C), high‐density lipoprotein cholesterol (HDL‐C), triglycerides (TG), uric acid, urine albumin, and creatinine were measured in the NHANES laboratory. The estimated glomerular filtration rate (eGFR) was calculated via the Chronic Kidney Disease Epidemiology Collaboration (CKD‐EPI) equation (Levey et al. 2009).

### 2.4 Primary Outcomes

The primary outcomes of this study include all‐cause mortality, CVD mortality, and cancer mortality. Mortality data as of December 31, 2019, were obtained through linkage with the National Death Index. The underlying causes of death were classified according to the International Classification of Diseases 10th Revision (ICD‐10).

### 2.5 Statistical Analysis

All statistical analyses were conducted using R software (version 4.4.3) and accounted for the complex, multistage probability sampling design of NHANES by incorporating the appropriate survey weights, strata, and primary sampling units (PSUs). This ensures that our findings are nationally representative of the U.S. non‐institutionalized civilian population.

Baseline characteristics of the study population were presented across quartiles of serum calcium. For categorical variables, we reported the unweighted sample counts (*n*) and survey‐weighted percentages (%). For continuous variables, we presented the survey‐weighted medians and interquartile ranges (IQRs). Comparisons between groups were performed using survey‐design‐based tests: the Wilcoxon rank‐sum test for continuous variables and the chi‐square test with Rao & Scott's second‐order correction for categorical variables. Mortality rates, calculated per 1000 person‐years with corresponding 95% confidence intervals (CIs), were estimated using a survey‐weighted Poisson regression model.

To evaluate the association between albumin‐adjusted serum calcium levels and mortality, we employed survey‐weighted Cox proportional hazards regression models to calculate hazard ratios (HRs) and 95% CIs. The R package “survey” was used for these analyses. We constructed three sequential models: Model 1 was adjusted for demographic factors (age, sex, race). Model 2 was further adjusted for lifestyle factors (BMI, drinking status, smoking status, education level, and family poverty income ratio). Model 3 (the fully adjusted model) included all covariates from Model 2 plus key clinical factors: duration of diabetes, diabetes medication use, fasting glucose, eGFR, uric acid, the urine albumin‐to‐creatinine ratio, and hypertension status. The proportional hazards assumption for the final Cox models was tested using Schoenfeld residuals, and no violations were detected (all *p* > 0.05), supporting the appropriateness of the model.

The potentially non‐linear dose–response relationship between continuous serum calcium concentration and mortality was modeled using survey‐weighted restricted cubic splines (RCS) with four knots, derived from the fully adjusted Cox models. The reference value for HR was set at the nadir of the curve (point of lowest risk). The “rms” and “survey” packages in R were utilized for the RCS analysis.

This analysis involved fitting a survey‐weighted linear regression model for the mediator and a survey‐weighted logistic regression model for the binary outcome. The significance of the indirect effect was assessed using 1000 bootstrap resamples to generate confidence intervals. Furthermore, we performed subgroup analyses based on serum calcium levels using survey‐weighted Cox regression models via the “jstable” R package, adjusting for multiple potential confounders. For baseline characteristics and other secondary analyses, a two‐sided *p* value < 0.05 was considered statistically significant. Given that we analyzed three primary mortality outcomes (all‐cause, CVD, and cancer), we applied a Bonferroni correction to account for multiple comparisons in our main Cox regression analyses. Consequently, a *p* value < 0.0167 (0.05/3) was considered statistically significant for these primary outcome analyses.

## 3 Results

### 3.1 Baseline Characteristics

The baseline demographic and clinical characteristics of the study population are summarized in Table 1 and Table S1. The cohort of T2DM consisted of 8131 participants, with a median age of 61.00 years and a median BMI of 31.63 kg/m^2^ (Table 1). By comparison, the cohort without diabetes consisted of 44,100 participants, with a median age of 43.00 years and a median BMI of 27.07 kg/m^2^ (Table S1). The participants in both cohorts with lower serum calcium levels were more likely to be younger, predominantly male, have a higher poverty‐income ratio, consume more alcohol, and have a lower BMI. Additionally, within the cohort with T2DM, those with lower serum calcium levels were more likely to not be taking diabetes medication, have a shorter duration of diabetes, and have a lower incidence of hypertension than those with higher serum calcium levels (all *p* < 0.05).

**TABLE 1:** Baseline characteristics and biomarkers by quartiles of albumin‐adjusted serum calcium in participants with T2DM.

Characteristic	Overall	Serum adjusted calcium				*p* ^c^
		Q1	Q2	Q3	Q4	
	*n* = 8131^a^	*n* = 1974^a^	*n* = 2030^a^	*n* = 2074^a^	*n* = 2053^a^	
	*N* = 247,095,912^b^	*N* = 62,040,092^b^	*N* = 63,461,849^b^	*N* = 62,511,129^b^	*N* = 59,082,840^b^	
Age, year	61.00 (50.00, 70.00)	58.00 (48.00, 68.00)	61.00 (50.00, 70.00)	61.00 (51.00, 70.00)	62.00 (53.00, 72.00)	< 0.001
*Gender*						
Female	3905 (48%)	718 (37%)	871 (43%)	1057 (52%)	1259 (63%)	< 0.001
Male	4226 (52%)	1256 (63%)	1159 (57%)	1017 (48%)	794 (37%)	
*Race*						
Mexican American	1684 (9.5%)	560 (13%)	442 (9.7%)	384 (8.6%)	298 (6.1%)	< 0.001
Other Hispanic	756 (6.1%)	184 (5.6%)	195 (6.3%)	207 (6.6%)	170 (5.7%)	
Non‐Hispanic White	2949 (62%)	720 (63%)	769 (64%)	741 (61%)	719 (60%)	
Non‐Hispanic Black	1970 (14%)	275 (7.9%)	409 (12%)	569 (16%)	717 (21%)	
Other/multiracial	772 (8.6%)	235 (10%)	215 (8.8%)	173 (8.3%)	149 (7.1%)	
*Education*						
< 9th	1646 (11%)	472 (13%)	397 (10%)	388 (11%)	389 (12%)	0.080
9–11th	1424 (15%)	320 (13%)	347 (14%)	363 (14%)	394 (17%)	
High school	1856 (26%)	420 (24%)	456 (25%)	502 (27%)	478 (27%)	
College or above	3190 (48%)	759 (50%)	827 (50%)	820 (49%)	784 (45%)	
Poverty‐income ratio	2.43 (1.27, 4.40)	2.64 (1.33, 4.62)	2.50 (1.34, 4.54)	2.38 (1.29, 4.50)	2.08 (1.16, 4.07)	0.001
*Smoking status*						
Never smoker	4010 (49%)	961 (49%)	994 (47%)	1051 (51%)	1004 (48%)	0.6
Former smoker	2781 (35%)	728 (35%)	687 (36%)	681 (33%)	685 (34%)	
Current smoker	1309 (16%)	279 (16%)	340 (17%)	333 (15%)	357 (17%)	
*Drinking status*						
Non‐drinker	2046 (32%)	417 (27%)	461 (29%)	562 (33%)	606 (38%)	< 0.001
1–5 drinks/month	2932 (53%)	746 (53%)	746 (53%)	736 (54%)	704 (50%)	
5–10 drinks/month	267 (4.8%)	63 (5.6%)	63 (4.2%)	72 (4.6%)	69 (4.9%)	
10+ drinks/month	559 (11%)	153 (14%)	173 (14%)	125 (7.9%)	108 (7.5%)	
BMI	31.63 (27.60, 37.00)	31.16 (27.27, 35.80)	31.42 (27.61, 36.27)	31.59 (27.78, 37.28)	32.75 (27.82, 38.61)	< 0.001
*Medication*						
No insulin or pills	1337 (23%)	387 (29%)	340 (25%)	336 (21%)	274 (17%)	< 0.001
Only diabetes pills	3671 (56%)	859 (55%)	884 (56%)	934 (57%)	994 (58%)	
Any insulin use	1374 (20%)	250 (16%)	321 (19%)	364 (21%)	439 (25%)	
Duration of diabetes, year	8.00 (3.00, 15.00)	6.00 (3.00, 13.00)	8.00 (3.00, 15.00)	8.00 (3.00, 15.00)	9.00 (3.00, 16.00)	0.002
Stroke	701 (7.8%)	150 (6.5%)	174 (8.1%)	173 (7.8%)	204 (9.0%)	0.13
Congestive heart failure	728 (8.2%)	161 (7.0%)	158 (7.7%)	187 (9.2%)	222 (9.0%)	0.2
Coronary heart disease	817 (11%)	197 (9.8%)	212 (11%)	199 (11%)	209 (11%)	0.6
Angina	585 (7.8%)	138 (6.8%)	145 (7.6%)	152 (8.1%)	150 (9.0%)	0.3
Heart attack	851 (10%)	193 (9.2%)	200 (10.0%)	219 (11%)	239 (11%)	0.4
Hypertension	5692 (68%)	1257 (62%)	1385 (66%)	1468 (69%)	1582 (76%)	< 0.001
Glucose, mmol/L	7.4 (6.5, 9.2)	7.3 (6.5, 8.7)	7.4 (6.6, 9.3)	7.4 (6.5, 9.4)	7.5 (6.3, 10.0)	0.095
Insulin, pmol/L	93 (55, 150)	91 (51, 144)	90 (54, 149)	90 (56, 143)	99 (59, 181)	0.12
HbA1c, %	6.70 (6.00, 7.70)	6.50 (5.90, 7.30)	6.60 (6.00, 7.50)	6.80 (6.20, 7.90)	6.80 (6.10, 8.00)	< 0.001
Total cholesterol, mmol/L	4.76 (4.06, 5.61)	4.63 (4.01, 5.41)	4.76 (4.06, 5.53)	4.78 (4.06, 5.61)	4.89 (4.14, 5.82)	< 0.001
HDL, mmol/L	1.16 (0.98, 1.40)	1.11 (0.93, 1.34)	1.16 (0.98, 1.40)	1.16 (1.00, 1.40)	1.22 (1.01, 1.47)	< 0.001
LDL, mmol/L	2.66 (2.07, 3.34)	2.64 (2.04, 3.31)	2.64 (2.10, 3.23)	2.64 (2.04, 3.31)	2.74 (2.04, 3.60)	0.4
Triglycerides, mmol/L	1.59 (1.10, 2.26)	1.56 (1.07, 2.26)	1.56 (1.10, 2.24)	1.57 (1.06, 2.30)	1.66 (1.14, 2.25)	0.6
Uric acid, umol/L	333 (280, 399)	333 (280, 393)	333 (280, 393)	333 (274, 405)	345 (286, 410)	0.005
eGFR, mL/min/1.73 m^2^	72 (55, 93)	74 (58, 96)	74 (57, 91)	72 (55, 93)	70 (53, 91)	0.002
Urine albumin/creatinine, mg/g	12 (6, 34)	11 (6, 26)	11 (6, 33)	12 (6, 34)	15 (7, 49)	< 0.001

*Note:* Data are presented as Median (interquartile range, IQR) for continuous variables and *n* (%) for categorical variables. Percentages (%) are survey‐weighted. Participants were divided into four groups based on the quartiles of their albumin‐adjusted serum calcium levels: Q1 (1.560–2.270 mmol/L), Q2 (2.270–2.325 mmol/L), Q3 (2.325–2.385 mmol/L), and Q4 (2.385–3.225 mmol/L).

Abbreviations: BMI, body mass index; eGFR, estimated glomerular filtration rate; HbA1c, glycated hemoglobin A1c.

^a^ Unweighted sample size (*n*).

^b^ Survey‐weighted population estimate (*N*), representing the non‐institutionalized U.S. civilian population.

^c^ *p*‐values were calculated accounting for the complex survey design. The survey‐weighted Wilcoxon rank‐sum test was used for continuous variables, and the chi‐squared test with Rao & Scott's second‐order correction was used for categorical variables.

For serum biomarkers, lower serum calcium levels were significantly correlated with lower levels of HbA1c, TC, HDL‐C, uric acid, and higher eGFRs in participants with T2DM (all *p* < 0.05). In participants without diabetes, lower serum calcium levels were significantly correlated with lower insulin, HbA1c, TC, HDL‐C, LDL‐C, TG, uric acid, urine albumin‐to‐creatinine ratios, and higher eGFRs (all *p* < 0.05).

### 3.2 Mortality Rates by Cause of Death per 1000 Person‐Years

During a follow‐up of an average duration of 7.95 years in the cohort of 8131 participants with T2DM, a total of 2321 participants died. Mortality rates per 1000 person‐years were examined across quartiles of blood calcium concentration. The total mortality rate increased from 30.26 (Q1) to 46.94 (Q4) per 1000 person‐years. These findings indicate an overall increase in mortality risk with increasing blood calcium concentrations, particularly for cardiovascular diseases, where mortality rates rose from 8.25 (Q1) to 13.02 (Q4) per 1000 person‐years. Cancer was the second leading cause of death, with mortality rates ranging from 5.38 (Q1) to 7.94 (Q4) per 1000 person‐years (Table S2).

In the cohort of 44,100 participants without diabetes, a total of 5232 participants died during an average follow‐up duration of 10.26 years. The total mortality rate increased from 9.3 (Q1) to 15.95 (Q4) per 1000 person‐years. These findings also suggest an overall increase in mortality risk with higher blood calcium concentrations, particularly for diseases of the heart and cancer. Mortality rates rose from 2.4 (Q1) to 3.99 (Q4) per 1000 person‐years due to heart disease and from 2.33 (Q1) to 3.46 (Q4) per 1000 person‐years due to cancer. However, these mortality rates are unadjusted, and further analysis controlling for potential confounding factors is needed (Table S2).

### 3.3 Multivariate‐Adjusted Cox Proportional Hazards Analyses Stratified by Serum Calcium Levels

Multivariate‐adjusted survival curves stratified by serum calcium levels are depicted in Figure S1. Specifically, as shown in Figure S1, in the cohort with T2DM, the survival curves demonstrated that participants with higher serum calcium levels likely exhibited lower survival probabilities than those with lower levels.

The detailed results of the multivariate‐adjusted Cox regression analyses are presented in Table 2. Higher serum calcium levels were consistently linked to increased all‐cause mortality rates, with the highest quartile (Q4) showing the highest mortality risk. Specifically, after full adjustment, the association remained significant (HR for Q3: 1.27, 95% CI: 0.97–1.67, *p* = 0.085; HR for Q4: 1.50, 95% CI: 1.15–1.96, *p* = 0.003). After adjustment for all covariates, elevated serum calcium levels remained an independent risk factor for mortality from CVD and cancer (Table 2).

**TABLE 2:** Cox regression analyses of serum calcium and mortality in cohorts with T2DM and without diabetes.

Characteristic	Serum adjusted calcium (T2DM)				Serum adjusted calcium (without diabetes)			
	Q1	Q2	Q3	Q4	Q1	Q2	Q3	Q4
*All‐cause mortality*								
Model 1	Ref	1.11 (0.94, 1.30); 0.2	1.21 (1.05, 1.40); 0.010	1.62 (1.38, 1.89); < 0.001	0.98 (0.86, 1.11); 0.7	1.09 (0.99, 1.19); 0.084	Ref	1.27 (1.16, 1.39); < 0.001
Model 2	Ref	1.15 (0.93, 1.42); 0.2	1.24 (1.01, 1.51); 0.040	1.63 (1.30, 2.04); < 0.001	1.14 (0.99, 1.30); 0.067	1.14 (1.02, 1.28); 0.023	Ref	1.31 (1.18, 1.45); < 0.001
Model 3	Ref	1.17 (0.90, 1.54); 0.2	1.27 (0.97, 1.67); 0.085	1.50 (1.15, 1.96); 0.003	1.14 (0.99, 1.31); 0.069	1.10 (0.97, 1.26); 0.15	Ref	1.29 (1.16, 1.44); < 0.001
*CVD mortality*								
Model 1	Ref	1.16 (0.81, 1.66); 0.4	1.40 (1.06, 1.84); 0.017	1.93 (1.42, 2.63); < 0.001	1.05 (0.86, 1.29); 0.6	1.09 (0.91, 1.30); 0.4	Ref	1.29 (1.09, 1.53); 0.003
Model 2	Ref	1.37 (0.90, 2.09); 0.14	1.58 (1.14, 2.20); 0.007	2.12 (1.42, 3.15); < 0.001	1.17 (0.90, 1.51); 0.2	1.14 (0.89, 1.47); 0.3	Ref	1.38 (1.12, 1.72); 0.003
Model 3	Ref	1.46 (0.85, 2.53); 0.2	1.70 (1.04, 2.78); 0.034	1.84 (1.06, 3.19); 0.030	1.19 (0.91, 1.55); 0.2	1.06 (0.80, 1.41); 0.7	Ref	1.31 (1.05, 1.65); 0.019
*Cancer mortality*								
Model 1	Ref	1.06 (0.73, 1.55); 0.7	0.93 (0.65, 1.33); 0.7	1.76 (1.25, 2.48); 0.001	1.06 (0.84, 1.33); 0.6	1.10 (0.90, 1.34); 0.4	Ref	1.27 (1.03, 1.57); 0.024
Model 2	Ref	0.97 (0.62, 1.54); > 0.9	1.03 (0.66, 1.61); 0.9	1.88 (1.26, 2.82); 0.002	1.30 (1.02, 1.66); 0.037	1.11 (0.87, 1.42); 0.4	Ref	1.28 (1.03, 1.57); 0.023
Model 3	Ref	0.85 (0.44, 1.65); 0.6	1.25 (0.66, 2.37); 0.5	2.32 (1.28, 4.20); 0.006	1.26 (0.98, 1.63); 0.072	1.06 (0.81, 1.39); 0.7	Ref	1.28 (1.03, 1.60); 0.028

*Note:* Model 1: Adjusted for age, sex, and race. Model 2: Built upon Model 1 by further adjusting for BMI, drinking status, smoking status, education level, and the family poverty income ratio. Model 3: Included all the covariates from Model 2 and additionally adjusted for duration of diabetes, diabetes medication use, fasting glucose, estimated glomerular filtration rate, uric acid, the urine albumin‐to‐creatinine ratio, and the presence of comorbidities.

In the cohort without diabetes, a U‐shaped association was observed between serum calcium levels and mortality rates, with the third quartile (Q3) having the lowest mortality risk (Table 2). Specifically, in Model 3, both lower (Q1 and Q2) and higher (Q4) serum calcium levels were associated with higher all‐cause mortality rates than the Q3 group (the reference group). The HRs for Q1, Q2, and Q4 were as follows: Q1: 1.14 (95% CI: 0.99–1.31; *p* = 0.069), Q2: 1.10 (95% CI: 0.97–1.26; *p* = 0.15), and Q4: 1.29 (95% CI: 1.16–1.44; *p* < 0.001). For cause‐specific mortality, the HR for CVD mortality in Q4 was 1.31 (95% CI: 1.05–1.65; *p* = 0.019), and for cancer mortality, it was 1.28 (95% CI: 1.03–1.60; *p* = 0.028). However, after applying the Bonferroni correction for multiple comparisons (significance threshold *p* < 0.0167), these associations were no longer statistically significant. These findings suggest a U‐shaped relationship, with increased mortality risk at both ends of the serum calcium distribution. However, the categorization may have obscured the non‐linear and subtle changes in survival risk that occur within the middle ranges of blood calcium levels.

### 3.4 Dose–Response Relationship Between Serum Calcium Concentration and Mortality

**FIGURE 2:**
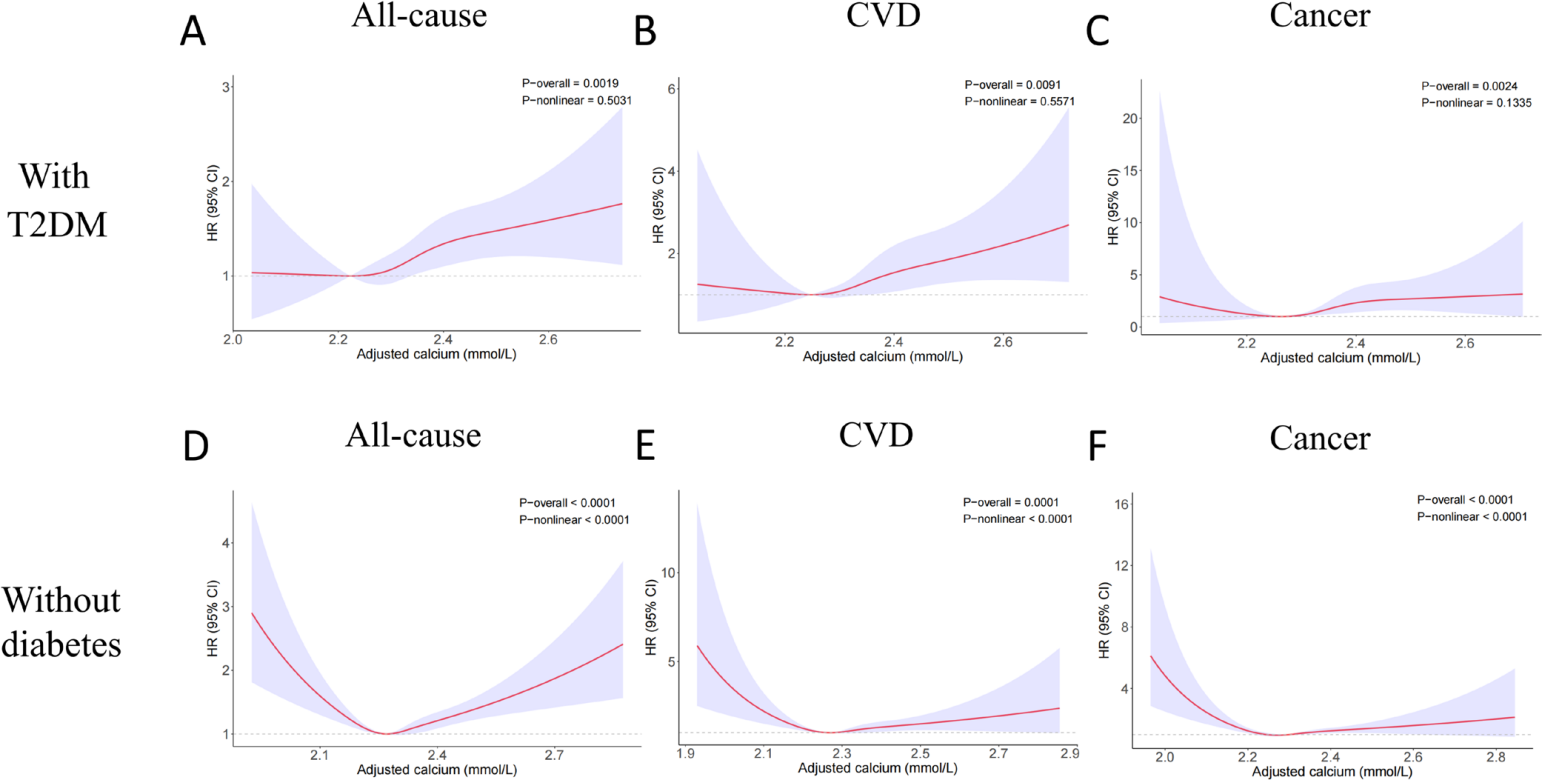
Dose–response relationships between the serum calcium concentration and mortality. Unrestricted cubic splines analysis of the dose–response relationship between the serum calcium concentration and (A) all‐cause mortality, (B) CVD mortality, and (C) cancer mortality in the cohort with T2DM and (D) all‐cause mortality, (E) CVD mortality, and (F) cancer mortality in the cohort without diabetes. Analyses were adjusted for multiple potential confounders via a Cox regression model with a complex survey design. Confounders included age, sex, race, education level, smoking and alcohol use, BMI, poverty‐income ratio, hypertension, fasting glucose, eGFR, uric acid, diabetes duration (for the T2DM cohort), the urine albumin‐to‐creatinine ratio, and medication use (for the T2DM cohort).

To accurately evaluate the dose–response relationship between the serum calcium concentration and mortality, we conducted unrestricted cubic spline analysis (Figure 2). For the cohort with T2DM, the relationship between the serum calcium concentration and all‐cause mortality exhibited a J‐shaped characteristic, and the *p* value for non‐linearity was not statistically significant (*p* = 0.5031, Figure 2A). Below approximately 2.3 mmol/L, the relationship was relatively stable, with the HR confidence interval including 1, indicating no significant effect on mortality. However, at higher concentrations, there was a significant increase in the HR. Similar trends were observed for CVD (Figure 2B) and cancer mortality (Figure 2C).

In contrast, the cohort without diabetes demonstrated a U‐shaped relationship, with statistically significant non‐linearity (*p* < 0.05) in the associations between serum calcium concentration and all‐cause mortality (Figure 2D), CVD mortality (Figure 2E), and cancer mortality (Figure 2F). For all‐cause mortality, the lowest HR was found at a serum calcium concentration of 2.27 mmol/L, with HRs increasing both above and below this concentration, particularly at lower serum calcium levels (Figure 2D). These findings indicate that in the population without diabetes, there is an optimal range for serum calcium levels that minimizes mortality risk.

### 3.5 Subgroup Analysis

Figure S2 illustrates the findings of a subgroup analysis through the use of a forest plot. The subgroups were categorized on the basis of serum calcium levels: the Q1 quartile represents the low calcium group, Q2 and Q3 form the moderate calcium group, and Q4 represents the high calcium group. Among the participants with T2DM, those who were over 60 years old and male had significantly higher all‐cause mortality rates when their serum calcium levels were in the high range (Q4) than when they were in the moderate range (Q2 and Q3) (*p* < 0.05). Conversely, there was no notable difference in all‐cause mortality rates among the subgroups when serum calcium levels were in the low range (Q1) compared with the moderate range (Figure S2A). Regarding CVD mortality (Figure S2B), there were no significant differences observed across the subgroups, as the serum calcium levels varied. In people who were older than 80 years, males were at greater risk of cancer mortality when their serum calcium concentration was elevated (Figure S2C).

Among participants without diabetes, the all‐cause mortality risk of participants younger than 80 years was slightly greater than that of participants over 80 years as the serum calcium concentration increased. Additionally, participants aged 60–79 years had a slightly greater all‐cause mortality risk than those over 80 years and under 60 years as the serum calcium concentration decreased (Figure S2D). Regarding CVD mortality, individuals younger than 60 years, specifically males, were more prone to an increased risk of CVD mortality when serum calcium concentration was elevated (Figure S2E). Concerning cancer mortality, black participants faced a higher risk of cancer mortality when serum calcium concentrations were elevated (Figure S2F). These findings suggest that specific demographic factors, including age, ethnicity, and sex, influence the association between serum calcium levels and mortality risk. Elevated serum calcium levels appear to be more detrimental in certain subgroups.

### 3.6 Mediation Analysis

To analyze the mechanisms potentially linking serum calcium to the mortality of participants with T2DM, we performed mediation analysis. After adjustment, all direct effect ORs for serum calcium were > 1 (all *p* < 0.001), validating its direct risk effect on the survival of participants with diabetes (Table 3). For all‐cause mortality, the indirect effects of serum calcium levels, mediated by the eGFR, uric acid, and the urine albumin‐to‐creatinine ratio (ACR), accounted for 12.61%, 4.62%, and 7.11% of the total effect, respectively (all *p* < 0.001). Total cholesterol mediated 4.29% of the association between serum calcium and all‐cause mortality.

**TABLE 3:** Mediation effect of serum calcium levels on all‐cause mortality in participants with diabetes.

	Indirect effect	Indirect *p*	Direct effect	Direct *p*	Prop.
Glucose, mmol/L	1.0001	0.32	1.0027	< 0.001	0.0185
HbA1c, %	1.0001	0.32	1.0055	< 0.001	0.0221
Insulin, pmol/L	1	0.72	1.0069	< 0.001	−0.0091
Total cholesterol, mmol/L	1.0003	0.02	1.006	< 0.001	0.0429
Triglycerides, mmol/L	1.0001	0.28	1.0029	< 0.001	0.0164
LDL, mmol/L	1	0.42	1.0023	< 0.001	−0.0156
HDL, mmol/L	1	0.7	1.006	< 0.001	−0.0007
eGFR, mL/min/1.73 m^2^	1.0007	0	1.0047	< 0.001	0.1261
Uric acid, mg/dL	1.0003	0	1.0053	< 0.001	0.0462
Urine albumin/creatinine, mg/g	1.0004	0	1.0055	< 0.001	0.0711

*Note:* The analysis included multivariate adjustments for age, sex, race, BMI, drinking status, smoking status, education level, family poverty income ratio, duration of diabetes, diabetes medication use, fasting glucose, eGFR, uric acid, the urine albumin‐to‐creatinine ratio, and the presence of comorbidities.

For CVD mortality, the indirect effects of serum calcium, mediated by eGFR, uric acid, and urine ACR, accounted for 16.65%, 6.76%, and 5.11% of the total effect, respectively (all *p* < 0.001). Total cholesterol mediated 8.68% of the association between serum calcium and CVD mortality (Table 3). For cancer mortality, we found no evidence that any serum biomarker mediates the relationship between serum calcium and cancer mortality (Table 3). These findings suggest that the increased serum calcium levels leading to increased cancer mortality may be due primarily to a direct effect. These findings indicate that, in addition to the direct effect, the relationship between serum calcium levels and mortality in participants with diabetes is partially mediated by markers of renal function (eGFR, urine ACR, uric acid) and total cholesterol for both all‐cause mortality and CVD mortality.

## 4 Discussion

In this study, our objective was to investigate the relationships between serum calcium levels and all‐cause, CVD, and cancer mortality rates among two separate adult populations: those with T2DM and those without diabetes. By analyzing data from the NHANES, we identified unique patterns of associations between serum calcium levels and mortality risk in these two groups.

For the cohort with T2DM, our results revealed a J‐shaped relationship between serum calcium concentration and mortality risk. Specifically, mortality risk remained relatively stable and low at lower calcium levels, but there was a marked increase in risk as calcium levels rose. Conversely, the cohort without diabetes demonstrated a U‐shaped relationship, suggesting that both lower and higher serum calcium levels are linked with higher mortality risk, with an optimal range in the middle where the risk is lowest.

These differential patterns underscore a fundamental nutritional principle: optimal nutrient thresholds are context‐dependent, particularly in metabolic diseases. For individuals with diabetes, maintaining serum calcium levels within a relatively lower but normal range could reduce the risk of adverse health outcomes, especially in those who have known risk factors for cardiovascular disease or cancer. On the other hand, for individuals without diabetes, maintaining serum calcium levels within a moderate range appears to be more beneficial for overall health optimization. We recommend incorporating the measurement of serum calcium levels into routine annual health check‐ups for people with or without diabetes, thereby optimizing health outcomes and reducing the risk of adverse events. This supports the concept of personalized nutrient safety margins in clinical nutrition—a key focus for precision nutrition initiatives.

To our knowledge, this is the first study to differentiate the relationships between serum calcium levels and mortality in participants with and without T2DM. Our findings partially align with a prior study investigating the relationship between serum calcium levels and cardiovascular disease mortality within two general population cohorts from the UK Biobank and the NHANES (Yang, Miao, et al. 2023). That study reported a U‐shaped association, which aligns with our findings in the cohort without diabetes. A separate study demonstrated that the regular consumption of calcium supplements is significantly correlated with an increased risk of cardiovascular disease events and mortality among individuals with diabetes, whereas no such association is observed in individuals without diabetes (Qiu et al. 2024). These findings indicate that healthcare providers should meticulously evaluate the potential adverse effects of calcium supplementation in relation to its anticipated benefits, especially in individuals with diabetes.

By delineating the relationship between serum calcium concentration and mortality in populations with or without diabetes, we provide valuable insights that can inform the development of tailored prevention and treatment strategies for different populations. These findings underscore the importance of personalized approaches to managing serum calcium levels on the basis of the presence or absence of T2DM and other comorbid conditions.

We further performed subgroup analysis, and the findings suggest that specific demographic factors, including age, ethnicity, and sex, influence the association between serum calcium levels and mortality risk. Notably, participants with diabetes over 60 years and males have significantly higher all‐cause mortality rates when serum calcium levels were elevated. These results underscore the need for targeted interventions aimed at helping older males with diabetes manage their serum calcium levels to potentially reduce all‐cause mortality.

To further elucidate the mechanisms potentially linking serum calcium to mortality in participants with diabetes, we performed a mediation analysis. This analysis validated the direct risk effect of serum calcium levels on survival status in participants with diabetes. High serum calcium concentrations are linked to artery calcification (Bolland et al. 2010; Montalcini et al. 2012; Rubin et al. 2007), a pathological process associated with many clinical implications, including atherosclerosis, chronic kidney disease, and diabetes (Jiang et al. 2017; Lin et al. 2018). Cardiovascular calcification is an independent risk factor and predictor of cardiovascular accidents and stroke (Hutcheson et al. 2016; Khan et al. 2020; Kramann et al. 2016; You et al. 2017). In addition, higher serum calcium levels are likely to lead to higher urinary calcium excretion (UCaE), which can contribute to renal function impairment (Taylor et al. 2017), thereby increasing mortality rates.

Studies have shown that hypocalcemia is particularly associated with mortality in cases of hemorrhagic stroke, heart failure, and ischemic heart disease (Kucukceylan et al. 2024). Various hypotheses have been proposed to elucidate the link between lower serum calcium levels and increased mortality rates (Denham et al. 2018). First, reduced calcium levels may result from an influx of calcium into cells, a process implicated in ischemic cell death (Goto et al. 2024). Second, both low and high serum calcium levels can disrupt neuromuscular stability (Yang, Kweon, et al. 2023). Additionally, hypocalcemia may co‐occur with malnutrition and immune dysfunction, which can increase the risk of infection (Wu et al. 2024).

In addition to its direct effects, the impact of serum calcium levels on the survival status of participants with diabetes is partly mediated through changes in renal function and total cholesterol, influencing both all‐cause mortality and CVD mortality. This finding aligns with previous research indicating that kidney function can modify the non‐linear relationship between calcium dyshomeostasis and CVD mortality (Yang, Kweon, et al. 2023). Therefore, monitoring renal function, uric acid, and lipid profiles may be beneficial in managing serum calcium levels and reducing mortality risk.

While our study provides valuable insights into the relationship between serum calcium levels and mortality, several limitations should be considered. First, the observational nature of the study design precludes establishing causality. Second, the NHANES data rely on self‐reported information, which may introduce bias. Third, residual confounding cannot be ruled out despite our efforts to adjust for multiple potential confounders. Fourth, our analysis of three distinct mortality outcomes raised the issue of multiple comparisons. To mitigate the risk of false‐positive findings, we employed a conservative Bonferroni correction, which adds robustness to our significant results. However, this also meant that some associations for cause‐specific mortality in the non‐diabetic cohort, while nominally significant, did not meet the adjusted threshold for statistical significance and should be interpreted with caution. Further mechanistic studies are needed to understand the underlying biological mechanisms linking serum calcium levels with mortality.

## 5 Conclusions

In conclusion, our study reveals distinct patterns of associations between serum calcium levels and mortality risk in individuals with T2DM and without diabetes. These findings underscore the importance of considering individual diabetic status when assessing the risk of mortality related to serum calcium levels and may guide future clinical practice and research in this area. This precision nutrition approach may mitigate preventable mortality while guiding targeted dietary interventions over universal supplementation.

## Author Contributions

Conceptualization: L.L. and X.R. Methodology: S.Y. Writing – original draft preparation: L.L. Writing – review and editing: S.L. All the authors have read and agreed to the published version of the manuscript.

## Ethics Statement

The studies involving humans were approved by the National Center for Health Statistics Ethics Review Board.

## Consent

Written informed consent was obtained from all study participants.

## Conflicts of Interest

The authors declare no conflicts of interest.

## Supporting information


**Figure S1:** fsn371034‐sup‐0001‐Supinfo.docx.
**Figure S2:** fsn371034‐sup‐0001‐Supinfo.docx.
**Table S1:** fsn371034‐sup‐0001‐Supinfo.docx.
**Table S2:** fsn371034‐sup‐0001‐Supinfo.docx.

## Data Availability

The data that support the findings of this study are available in National Center for Health Statistics (NCHS) at https://wwwn.cdc.gov/nchs/nhanes/. These data were derived from the following resources available in the public domain: National Health and Nutrition Examination Survey (NHANES), https://wwwn.cdc.gov/nchs/nhanes/.
