# Supplementary material for: Diabetes‐Specific Serum Calcium Thresholds for Mortality Risk: A NHANES Nutritional Epidemiology Study
Source: Food Sci Nutr. 2025 Sep 23;13(9):e71034. doi: 10.1002/fsn3.71034 (PMC12457720; doi:10.1002/fsn3.71034)
Supplement: Supplementary file 1 — Figure S1: fsn371034‐sup‐0001‐Supinfo.docx. Figure S2: fsn371034‐sup‐0001‐Supinfo.docx. Table S1: fsn371034‐sup‐0001‐Supinfo.docx. Table S2: fsn371034‐sup‐0001‐Supinfo.docx. [file FSN3-13-e71034-s001.docx]

**Figure S1**
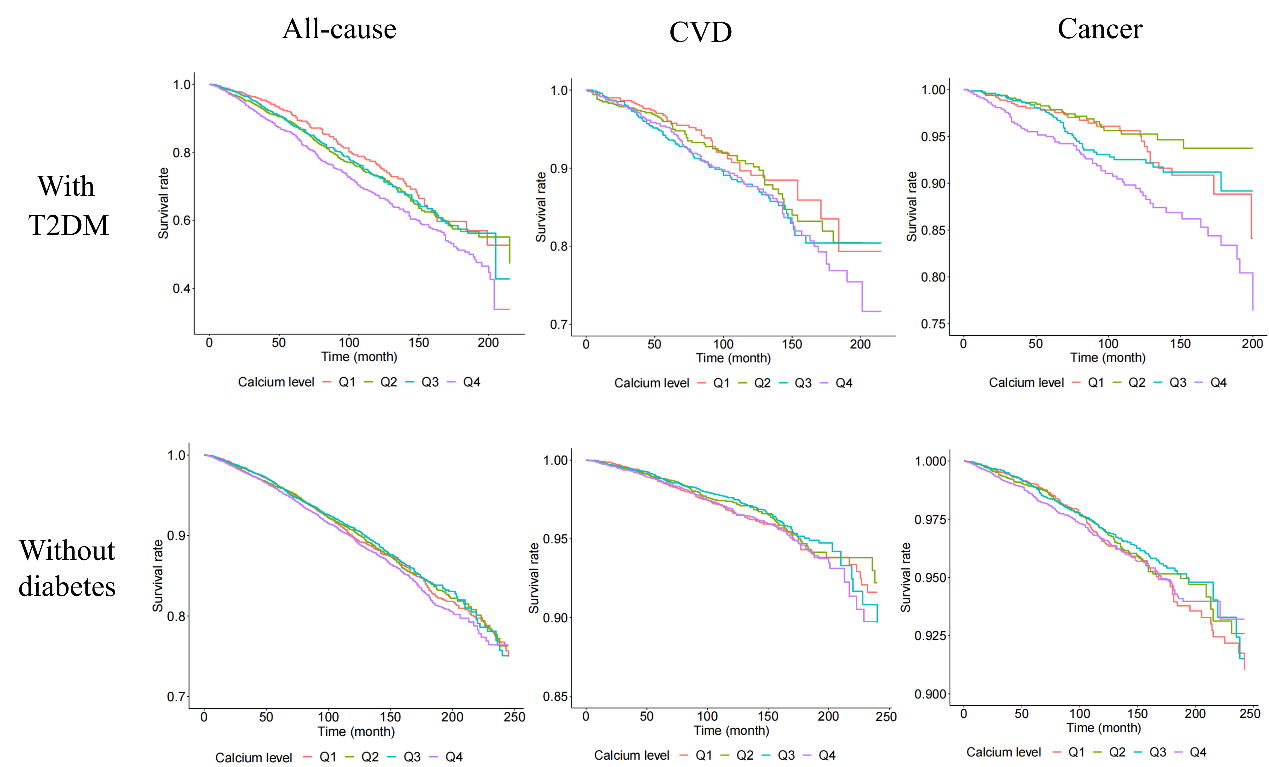


**Figure S1**: **Multivariate-adjusted survival curves stratified by serum calcium levels.** Survival curves stratified by serum calcium levels, adjusted for multiple potential confounders using a Cox regression model with a complex survey design. Confounders included age, sex, race, education level, smoking and alcohol use, BMI, poverty-income ratio, hypertension, fasting glucose, eGFR, uric acid, diabetes duration (for cohort with T2DM), urine albumin-to-creatinine ratio, and medication use (for cohort with T2DM).

**Figure S2**


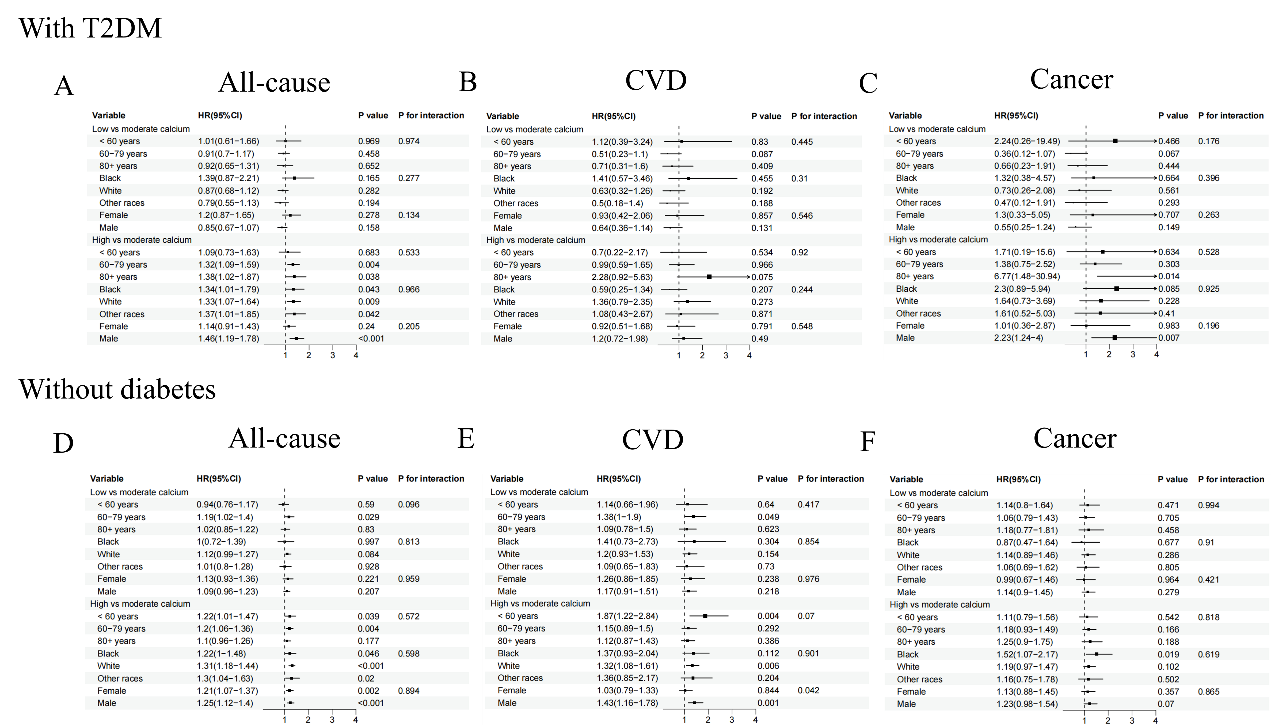


**Figure S2**: **Subgroup analysis of serum calcium concentration and mortality risk.** Forest plots showing the subgroup analysis of the association between serum calcium concentration and (A) all-cause mortality, (B) CVD mortality, and (C) cancer mortality in cohort with T2DM, and (D) all-cause mortality, (E) CVD mortality, and (F) cancer mortality in cohort without diabetes. Analyses were adjusted for multiple potential confounders using a Cox regression model. Confounders included age, sex, race, education level, smoking and alcohol use, BMI, poverty-income ratio, hypertension, fasting glucose, eGFR, uric acid, diabetes duration, urine albumin-to-creatinine ratio, medication use and cardiovascular diseases (congestive heart failure, coronary heart disease, angina, heart attack, stroke).

**Table S1** Baseline characteristics and biomarkers by quartiles of albumin-adjusted serum calcium in participants without diabetes.

|  | | **Serum adjusted calcium** | | | |  |
| --- | --- | --- | --- | --- | --- | --- |
| **Characteristic** | **Overall**  n=44100^1^  N=1856020483^2^ | **Q1**  n=10472^1^ N=467317638^2^ | **Q2**  n=11159^1^ N=492632886^2^ | **Q3**  n=11242^1^ N=475592271^2^ | **Q4**  n=11227^1^ N=420477687^2^ | ***P***  **Value**^3^ |
| Age, y | 43.00 (30.00, 56.00) | 40.00 (30.00, 52.00) | 42.00 (30.00, 54.00) | 43.00 (29.00, 57.00) | 47.00 (31.00, 62.00) | <0.001 |
| Gender |  |  |  |  |  | <0.001 |
| Female | 23,031 (52%) | 4,698 (45%) | 5,490 (49%) | 5,831 (52%) | 7,012 (63%) |  |
| Male | 21,069 (48%) | 5,774 (55%) | 5,669 (51%) | 5,411 (48%) | 4,215 (37%) |  |
| Race |  |  |  |  |  | <0.001 |
| Mexican American | 8,061 (8.2%) | 2,680 (11%) | 2,170 (8.8%) | 1,790 (7.2%) | 1,421 (5.8%) |  |
| Other Hispanic | 3,509 (5.7%) | 879 (6.1%) | 957 (5.8%) | 920 (5.7%) | 753 (5.0%) |  |
| Non-Hispanic White | 19,736 (69%) | 4,480 (69%) | 5,008 (70%) | 5,157 (70%) | 5,091 (68%) |  |
| Non-Hispanic Black | 8,807 (10%) | 1,123 (5.6%) | 1,865 (8.5%) | 2,510 (11%) | 3,309 (16%) |  |
| Other/multiracial | 3,987 (6.7%) | 1,310 (8.5%) | 1,159 (7.3%) | 865 (5.7%) | 653 (5.2%) |  |
| Education |  |  |  |  |  | <0.001 |
| <9th | 4,266 (5.0%) | 1,220 (5.6%) | 1,054 (4.7%) | 991 (4.7%) | 1,001 (5.2%) |  |
| 9-11th | 7,187 (12%) | 1,580 (11%) | 1,739 (11%) | 1,833 (12%) | 2,035 (13%) |  |
| High school | 10,596 (24%) | 2,311 (22%) | 2,562 (23%) | 2,830 (26%) | 2,893 (26%) |  |
| College or above | 21,866 (59%) | 5,306 (61%) | 5,772 (62%) | 5,541 (58%) | 5,247 (55%) |  |
| Poverty-income ratio | 3.02 (1.49, 5.00) | 3.18 (1.58, 5.00) | 3.18 (1.57, 5.00) | 2.95 (1.46, 5.00) | 2.70 (1.36, 4.64) | <0.001 |
| Smoking status |  |  |  |  |  | <0.001 |
| Never smoker | 23,038 (55%) | 5,716 (56%) | 5,893 (55%) | 5,824 (55%) | 5,605 (53%) |  |
| Former smoker | 9,333 (23%) | 2,293 (24%) | 2,319 (23%) | 2,285 (22%) | 2,436 (24%) |  |
| Current smoker | 8,883 (22%) | 1,921 (20%) | 2,304 (22%) | 2,330 (23%) | 2,328 (24%) |  |
| Drinking status |  |  |  |  |  | <0.001 |
| Non-drinker | 7,659 (20%) | 1,548 (17%) | 1,823 (18%) | 1,946 (20%) | 2,342 (26%) |  |
| 1-5 drinks/month | 15,195 (50%) | 3,638 (49%) | 3,985 (50%) | 3,896 (50%) | 3,676 (49%) |  |
| 5-10 drinks/month | 2,774 (11%) | 727 (12%) | 746 (11%) | 736 (11%) | 565 (8.7%) |  |
| 10+ drinks/month | 5,030 (20%) | 1,328 (22%) | 1,340 (21%) | 1,245 (19%) | 1,117 (16%) |  |
| BMI | 27.07 (23.61, 31.37) | 26.59 (23.30, 30.49) | 26.98 (23.50, 31.10) | 27.34 (23.80, 31.67) | 27.59 (23.94, 32.30) | <0.001 |
| Stroke | 1,118 (2.0%) | 207 (1.7%) | 230 (1.5%) | 259 (2.0%) | 422 (3.1%) | <0.001 |
| Congestive heart failure | 838 (1.5%) | 181 (1.2%) | 177 (1.3%) | 190 (1.5%) | 290 (2.2%) | <0.001 |
| Coronary heart disease | 1,197 (2.4%) | 242 (1.8%) | 275 (2.3%) | 301 (2.5%) | 379 (3.1%) | <0.001 |
| Angina | 817 (1.7%) | 156 (1.3%) | 177 (1.4%) | 206 (1.7%) | 278 (2.4%) | <0.001 |
| Heart attack | 1,265 (2.4%) | 260 (1.9%) | 290 (2.3%) | 294 (2.2%) | 421 (3.3%) | <0.001 |
| Hypertension | 14,053 (30%) | 2,774 (24%) | 3,264 (27%) | 3,580 (30%) | 4,435 (38%) | <0.001 |
| Glucose, mmol/L | 5.38 (5.02, 5.74) | 5.33 (5.00, 5.72) | 5.38 (5.05, 5.73) | 5.38 (5.05, 5.77) | 5.38 (5.01, 5.77) | 0.3 |
| Insulin, pmol/L | 53 (35, 84) | 50 (34, 77) | 53 (34, 83) | 53 (35, 85) | 56 (37, 90) | <0.001 |
| HbA1c, % | 5.30 (5.10, 5.60) | 5.30 (5.00, 5.50) | 5.30 (5.10, 5.60) | 5.40 (5.10, 5.60) | 5.40 (5.10, 5.60) | <0.001 |
| Total cholesterol, mmol/L | 4.99 (4.32, 5.72) | 4.81 (4.19, 5.48) | 4.91 (4.27, 5.64) | 5.07 (4.40, 5.77) | 5.20 (4.53, 5.95) | <0.001 |
| HDL, mmol/L | 1.32 (1.09, 1.63) | 1.29 (1.06, 1.58) | 1.32 (1.09, 1.60) | 1.32 (1.09, 1.63) | 1.37 (1.11, 1.68) | <0.001 |
| LDL, mmol/L | 2.92 (2.38, 3.54) | 2.82 (2.30, 3.44) | 2.90 (2.35, 3.52) | 3.00 (2.43, 3.65) | 3.03 (2.46, 3.62) | <0.001 |
| Triglycerides, mmol/L | 1.14 (0.79, 1.68) | 1.06 (0.72, 1.58) | 1.12 (0.77, 1.64) | 1.16 (0.82, 1.72) | 1.24 (0.86, 1.83) | <0.001 |
| Uric Acid, umol/L | 315 (256, 369) | 309 (256, 369) | 309 (256, 369) | 315 (262, 375) | 315 (262, 375) | <0.001 |
| eGFR, mL/min/1.73m^2^ | 86 (69, 104) | 89 (72, 108) | 86 (70, 105) | 84 (68, 102) | 84 (66, 101) | <0.001 |
| Urine albumin/creatinine, mg/g | 6 (4, 10) | 6 (4, 10) | 6 (4, 10) | 6 (4, 10) | 7 (4, 12) | <0.001 |

Data are presented as Median (interquartile range, IQR) for continuous variables and n (%) for categorical variables. Percentages (%) are survey-weighted.

Abbreviations: BMI, body mass index; eGFR, estimated Glomerular Filtration Rate; HbA1c, glycated hemoglobin A1c.

Participants were divided into four groups based on the quartiles of their albumin-adjusted serum calcium levels: Q1 (1.590-2.245 mmol/L), Q2 (2.245-2.300 mmol/L), Q3 (2.300-2.355 mmol/L), Q4 (2.355-3.660 mmol/L).

¹Unweighted sample size (n).

²Survey-weighted population estimate (N), representing the non-institutionalized U.S. civilian population.

³P-values were calculated accounting for the complex survey design. The survey-weighted Wilcoxon rank-sum test was used for continuous variables, and the chi-squared test with Rao & Scott's second-order correction was used for categorical variables

**Table S2.** Mortality rates by cause of death per 1000 person-years in participants with T2DM or without diabetes.

| **Cause of death** | **With T2DM** | | | |  | **Without diabetes** | | | |
| --- | --- | --- | --- | --- | --- | --- | --- | --- | --- |
|  | **Q1** | **Q2** | **Q3** | **Q4** |  | **Q1** | **Q2** | **Q3** | **Q4** |
| Diseases of heart | 8.25 (6.88, 9.75) | 8.93 (7.51, 10.48) | 10.61 (9.09, 12.26) | 13.02 (11.29, 14.88) |  | 2.4 (2.11, 2.71) | 2.41 (2.12, 2.7) | 2.48 (2.19, 2.78) | 3.99 (3.63, 4.36) |
| Cancer | 5.38 (4.25, 6.63) | 5.89 (4.72, 7.2) | 5.67 (4.57, 6.89) | 7.94 (6.57, 9.43) |  | 2.33 (2.04, 2.63) | 2.5 (2.21, 2.8) | 2.59 (2.31, 2.9) | 3.46 (3.13, 3.8) |
| Chronic lower respiratory diseases | 1.38 (0.81, 2.06) | 1.49 (0.93, 2.17) | 1.28 (0.79, 1.95) | 2.17 (1.49, 2.98) |  | 0.51 (0.38, 0.66) | 0.53 (0.4, 0.69) | 0.7 (0.55, 0.87) | 0.96 (0.79, 1.14) |
| Accidents | 0.69 (0.31, 1.19) | 0.87 (0.43, 1.43) | 0.24 (0.06, 0.61) | 0.62 (0.25, 1.12) |  | 0.39 (0.28, 0.52) | 0.48 (0.35, 0.62) | 0.38 (0.27, 0.51) | 0.56 (0.43, 0.7) |
| Cerebrovascular diseases | 2 (1.31, 2.81) | 1.55 (0.99, 2.23) | 2.07 (1.4, 2.87) | 3.16 (2.36, 4.15) |  | 0.49 (0.36, 0.64) | 0.59 (0.45, 0.74) | 0.55 (0.42, 0.69) | 0.91 (0.74, 1.09) |
| Alzheimer’s disease | 0.75 (0.38, 1.31) | 0.81 (0.37, 1.36) | 1.16 (0.67, 1.77) | 1.18 (0.68, 1.8) |  | 0.27 (0.18, 0.38) | 0.47 (0.34, 0.61) | 0.38 (0.27, 0.51) | 0.79 (0.63, 0.97) |
| Diabetes mellitus | 2.56 (1.81, 3.44) | 2.85 (2.05, 3.78) | 3.35 (2.5, 4.33) | 4.77 (3.72, 5.95) |  | 0.06 (0.02, 0.12) | 0.11 (0.05, 0.19) | 0.14 (0.08, 0.23) | 0.06 (0.02, 0.12) |
| Influenza and pneumonia | 0.75 (0.38, 1.31) | 0.62 (0.25, 1.12) | 0.61 (0.24, 1.1) | 1.12 (0.62, 1.74) |  | 0.15 (0.08, 0.24) | 0.17 (0.1, 0.26) | 0.23 (0.15, 0.33) | 0.38 (0.27, 0.5) |
| Nephritis, nephrotic syndrome and nephrosis | 0.94 (0.5, 1.5) | 1.43 (0.87, 2.11) | 0.98 (0.55, 1.59) | 1.86 (1.24, 2.6) |  | 0.15 (0.08, 0.24) | 0.12 (0.05, 0.2) | 0.11 (0.05, 0.19) | 0.32 (0.22, 0.44) |
| All other causes | 7.56 (6.25, 9) | 7.13 (5.83, 8.56) | 8.84 (7.44, 10.37) | 11.1 (9.49, 12.84) |  | 2.55 (2.25, 2.87) | 2.71 (2.41, 3.03) | 3.03 (2.72, 3.36) | 4.53 (4.16, 4.93) |
| Total | 30.26 (27.57, 33.07) | 31.58 (28.85, 34.43) | 34.82 (32.02, 37.75) | 46.94 (43.65, 50.35) |  | 9.3 (8.73, 9.88) | 10.08 (9.49, 10.69) | 10.59 (10, 11.2) | 15.95 (15.23, 16.68) |

Participants in each cohort were divided into four groups based on the quartiles of their albumin-adjusted serum calcium levels respectively: cohort with T2DM: Q1 (1.560-2.270), Q2 (2.270-2.325), Q3 (2.325-2.385), Q4 (2.385-3.225); Cohort without diabetes: Q1 (1.590-2.245), Q2 (2.245-2.300), Q3 (2.300-2.355), Q4 (2.355-3.660).
